# No effect of novel exploration on the consolidation of extinction learning in human context conditioning

**DOI:** 10.1038/s41598-025-05235-2

**Published:** 2025-06-20

**Authors:** Thomas Agren, Johannes Björkstrand, Jörgen Rosén

**Affiliations:** 1 Department of Psychology, Uppsala University, Box 1225, 751 42 Uppsala, Sweden; 2 Department of Occupational Health, Psychology and Sports Science, University of Gävle, Gävle, Sweden; 3 Department of Psychology, Lunds University, Lund, Sweden; 4 Department of Clinical Neuroscience, Karolinska Institutet, Stockholm, Sweden

**Keywords:** Fear conditioning, Context conditioning, Memory consolidation, Behavioural tagging, Virtual reality, Skin conductance, Psychology, Consolidation, Extinction, Fear conditioning, Long-term memory

## Abstract

Animal research show that a novel exploration task performed shortly before a learning episode can strengthen hippocampal memory consolidation through behavioural tagging mechanisms. The aim of the present study was to conceptually translate behavioural tagging results to humans using a novel exploration task in virtual reality. Mimicking conditions for animal research, sixty participants underwent a context conditioning task in virtual reality to create a hippocampal-dependent fear memory. Twenty-four hours later, half of the participants performed a novel exploration task in virtual reality shortly before extinction learning the next day, and the other half performed a visual control task. Twenty-four hours after extinction learning, remaining fear responses were evaluated by a reinstatement procedure. Results showed that participants acquired context conditioning, but no effect of the novel exploration procedure on fear responses during reinstatement could be noted. Thus, the study did not conceptually translate the rodent results to humans; possible reasons for this, as well as future directions, are discussed.

**Supplementary Information:**

The online version contains supplementary material available at 10.1038/s41598-025-05235-2.

## Introduction

Accumulating evidence from animal research show that memory consolidation is working through behavioural tagging mechanisms^1^. However, studies translating these results from animal to human are largely missing. Hence, in order to examine the effects of these mechanisms on human memory, and to explore possible clinical applications in manipulating behavioural tagging mechanisms, it is necessary to find experimental setups capable of translating results from animal research to humans.

The behavioural tagging hypothesis states that the formation of memories depend on the creation of molecular learning tags inside neurons, which later guide plasticity-related proteins (PRP) to these sites for consolidation of memory to occur^2^. An event is not encoded to long-term memory (LTM) if sufficient PRPs are not produced, and this may happen to a myriad of minor events and sensations each day that are not memorable or novel. The tags are believed to last about 2 h, and PRPs created as the result of a novel experience last at least 1 h^3^. An interesting consequence of this is that the encoding of different events can interact. For example, events that might not have ended up as long-term memories (LTM) can use the PRPs of a later occurring novel event, and thus, retroactively be marked for long-term storage. This has an obvious function for survival, as it is important to remember the events preceding a dangerous incident in order to be able to avoid it in the future. In addition, events that follow an event that incites the production of PRPs can use the PRPs of that event to aid consolidation and transform into a LTM^4^.

A few studies have observed results in line with behavioural tagging in humans. An unrelated novel event (a short science class) improved the memory of a story when the novel event took place one hour before or after the reading of the story, but not when it took place 4 h before or after the reading^5^. Two studies reported the retroactive strengthening of memories that were later associated to punishment or reward, but not the strengthening of other memories present in the same learning session^6,7^. The first study used category conditioning. In category conditioning, participants are shown individual unique exemplars of categories (e.g. tools, animals) and while displaying the exemplars of one of these categories, sometimes an electric shock is delivered. Thus, the participants come to associate one of the categories with threat, similar to classical conditioning. Results showed that memory of exemplars from the category that was later associated with an electric shock was increased^6^. However, several attempts to replicate these results have failed^8^. The second study mentioned above was similar to the study using category conditioning, but instead of an electric shock, a monetary reward was used. Here, results also showed increased memory for exemplars from the category that was later associated with a reward^7^.

In rodents, novel exploration has been used to strengthen the consolidation of a variety of hippocampal-dependent tasks in line with the behavioural tagging hypothesis^1^, and novel exploration was instrumental in the first demonstrations of such results^4^. In line with this, exploration of a novel, but not a familiar, environment in virtual reality increased the memory of an unrelated word test in humans^9^. However, translations of using novel exploration to strengthen the consolidation of hippocampus-dependent memories are lacking. According to the behavioural tagging hypothesis, PRPs must be created in the same neural structure where the utilizing behavioural tags are situated^1^. This appears a natural condition, and there are several corroborating results^3^. Hence, the behavioural tagging of hippocampus-dependent memories appears a good starting point for translational studies. In addition, hippocampal-dependent memories are important for human fear and anxiety, and therefore of clinical interest^10^.

An interesting result from rodent research concerns the use of novel exploration to strengthen the consolidation of an extinction learning^11^. Rodents went through contextual fear conditioning, that is, they learned that a specific context was associated with a threat. Twenty-four hours later, rodents went through an extinction session, that is, they were exposed to the threatening context, but with the threat withheld, and learned that the context was now safe. Spatial exploration of an open field 1–2 h before the extinction training, or 1 h after the extinction training, led to strengthened consolidation and subsequently less fear when rodents were evaluated for remaining fear yet another 24 h later.

Translating these results to humans would not only provide an experimental set-up for the study of behavioural tagging, but also be of clinical interest within the framework of exposure treatment. Context conditioning can be achieved in humans using virtual reality (VR)^12^, thus a direct translation of this study can be attempted. However, the experimental conditions will differ in several ways from experimental work with rodents. In humans, it is customary to use a control condition during fear conditioning in order to control for non-specific physiological response, that is, a safe context (CTX−) to contrast the conditioned context (CTX+). Presentations of an electric shock in the CTX+ creates a context conditioned memory, that is, an association between CTX+ and threat, while no such association is created between CTX- and threat. In humans, context conditioning thus contains two things to learn, that CTX+ is associated with danger, and that CTX- is associated with safety. In theory, memory encoding of these two pieces of learned information could be affected by behavioural tagging mechanisms. However, during extinction, where threat responses is removed by exposure to CTX+ and CTX− without electric shocks, only CTX changes valence (from danger to safety) and it is assumed that when manipulating the memory of extinction learning, only the CTX+ learning will be affected. Remaining fear 24 h later can be tested by a reinstatement procedure, during which one or more presentations of the unconditioned stimuli (US) are used to revive the association between CTX + and threat^13^. Because the competition between the context conditioning and extinction memory result in the response to CTX+, this procedure can be used to measure extinction memory strength. Novel exploration can also be performed in VR^9^, but such a novel exploration will probably be qualitatively different than an open field exploration for rodents, which is also associated with a fear response^14^. Moreover, in human context conditioning it appears very common with a semi-instructed procedure, that is, that the participant is instructed to pay attention to, and discover, an association between the stimuli during conditioning. Sometimes subjective ratings are also used to aid fear learning, either online or in between acquisition blocks^12^. Naturally, these procedures are not used in rodent research. These differences between procedures in animal and human research may limit the translatability of results from animal research.

If an experimental setup could be found that enabled the study of behavioural tagging, especially of fear-related memories in humans, it would be a valuable tool to further our knowledge of the emergence and maintenance of fear memories and allow us to explore possible clinical applications of manipulating this process. Although category conditioning is an interesting avenue^6^, it would be preferable to study a specific associative memory between a stimulus and a physiological response, as is done in standard fear conditioning, and with an experimental setup more akin to a direct translation of rodent studies to humans.

Thus, the primary aim of this study was to attempt to affect the strength of extinction memory in humans by introducing a novel exploration 60 min before extinction training, in line with behavioural tagging results in rodents^11^. A secondary aim was to examine if our experimental setup managed to produce context conditioning, extinction, and reinstatement, without a semi-instructed approach, or the cognitive processes brought about by subjective ratings.

Sixty participants took part in the experiment at three sessions over three consecutive days, undergoing acquisition in session 1 and extinction in session 2. Sixty minutes before extinction in session 2, half of the participants explored a novel environment in VR, and the other half performed a 2D visual attention task. Extinction memory was then evaluated during reinstatement and re-extinction in session 3.

## Methods

### Participants

Sixty participants were recruited through advertisements on social media and billboards on Uppsala University campuses. All participants were at least 18 years old and fluent in Swedish. Exclusion criteria consisted of self-reported current psychiatric disorder, neurological condition, and current use of psychotropic medication. The participants were reimbursed with gift cards to a total value of 300 SEK (about 25 USD) for the three sessions. Six participants did not complete all three sessions. Four participants dropped out because of VR-related motion sickness, and one became ill between sessions. One participant was excluded because of equipment malfunction. Thus, the first session was completed by 58 participants (29 men, 29 women; age M = 26.16, SD = 7.13, novel exploration *N* = 29, control *N* = 29), the second session was completed by 55 participants (novel exploration *N* = 28, control *N* = 27), and all three sessions were completed by 54 participants (novel exploration *N* = 27, control *N* = 27). Electromyography (EMG) data was corrupted for some sessions, leaving the following number of participants for EMG analysis: Session 1, *N* = 54, Session 2, *N* = 49, Session 3, *N* = 50.

The study was approved by the Swedish Ethical Review Authority (2021–03596) and all research was performed in accordance with relevant guidelines and regulations. All participants gave their written informed consent.

### Materials

#### Virtual reality

Virtual reality presentations were controlled using an HTC Vive Pro (stereoscopic 3D images at 2880 × 1600 pixels and 110 degrees diagonal running at 90 Hz) head-mounted display. All environments were programmed using Unity 5.6.3 (Unity Technologies, http://www.unity3d.com). The context conditioning environment consisted of two rooms with a connecting corridor. The two rooms (kitchen and bedroom) were designed to be similar in colour and furniture, but still easy to tell apart. The corridor was empty with just a door in either end of it. The environment for novel exploration was an autumn park surrounded by walls. The size of the park was chosen so that you would have time to explore it, but not to so much to know all its details. A few oddball items were placed in the park (i.e. a giant apple, a giant banana, a bathtub). See Supplementary Fig. 1 for a display of the rooms and the park.

#### Psychophysiology

Skin conductance and startle responses were measured using a Biopac MP-150 system (BIOPAC Systems, Goleta, CA). Skin conductance was measured using two disposable electrodes prepared with isotonic electrolyte gel (EL503; BIOPAC Systems, Goleta, CA) fastened on the hypothenar eminence of the left hand. Startle responses were measured at 10,000 Hz using three cup electrodes filled with electrolyte gel, two fastened over the orbicularis oculi muscle under the left eye, and a third (ground) fastened on the forehead. Startle bursts were binaural bursts of white noise presented for fifty milliseconds. Fifteen millisecond electric shocks were administrated through electrodes (EL500; BIOPAC Systems, Goleta, CA) prepared with electrolyte gel and a voltage stimulator (STM200; BIOPAC Systems, Goleta, CA) to the right forearm.

#### Ratings and questionnaires

After each context conditioning phase, participant rated their subjective experience of fear and discomfort regarding the procedure (0 = no fear/discomfort, 100 = strongest imaginable fear/discomfort). After the acquisition phase, participants were asked if they had discovered some sort of pattern of when they received the shocks, and if so, describe that pattern. Participants also completed the Swedish version of the State-Trait Anxiety Inventory (STAI-T)^15^ and the Generalized Anxiety Disorder-7 (GAD-7)^16^ for measures of trait anxiety, and the Patient Health Questionnaire-9 (PHQ-9)^17^ for a measure of symptoms of depression. The questionnaire data was collected as part of a larger data collection on the associations between individual differences in context conditioning and symptoms of anxiety and depression. They will be pooled with further data and thus, will not be presented here.

#### Procedure

**Fig. 1:**
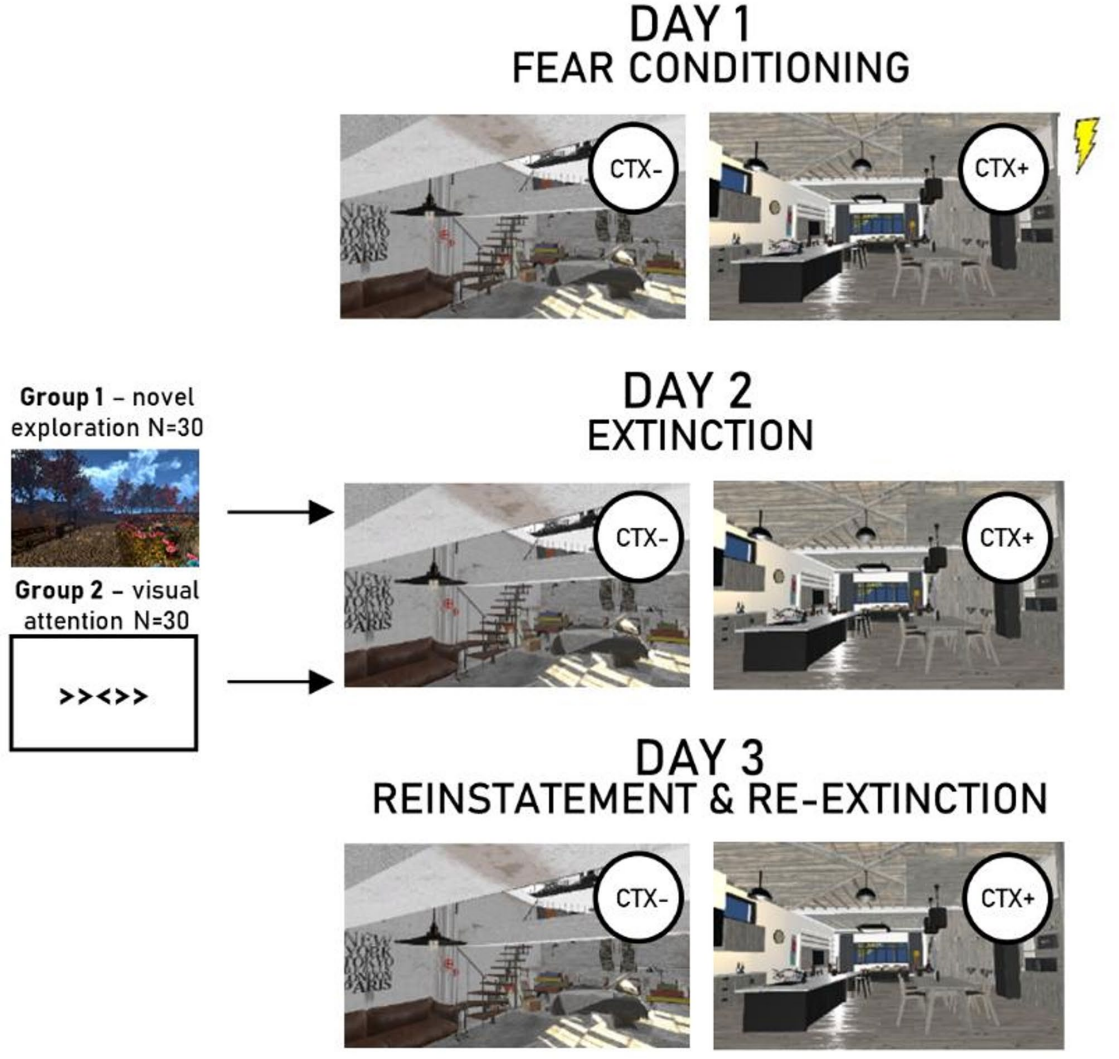
Experimental design.

The experiment took place over three sessions on three consecutive days, roughly 24 h apart, with acquisition on day 1, extinction on day 2, and reinstatement on day 3. Conditioned stimuli were the two rooms, one of which was associated with electric shocks (CTX+), and one was not (CTX−). The specific VR-contexts assigned to CTX+ and CTX− were counterbalanced across participants (See Fig. 1 for an overview of the experimental design).

#### Session 1

First, the participant gave their written informed consent to take part of the study. Then electrodes for measuring skin conductance and startle responses, as well as electrodes for delivering electric shocks, were fastened on the participants. The head-mounted display was then adjusted and calibrated in a simple VR environment consisting of a room with two coloured geometrical objects. Still in the calibration room, the volume of startle probes was then tested. If participants thought the volume was too loud, they had the opportunity to lower it. No participant lowered the volume so that it no longer produced startle responses. The level of the electric shocks was decided using a step-wise increase of shock strength until participants indicated that the sensation was uncomfortable, but acceptable.

Before the VR-session, participants were only given the instruction to observe their surroundings. During the VR-session, they sat on a chair and were passively led back and forth between the two different rooms on a pre-destined path that started and ended in the corridor. Each visit in a room lasted 30 s during which the participant was brought some distance into the room, stopped, turned, and then left the room. Between the room visits, 15 s was spent traversing the corridor. The participants visited each room 10 times. On four occasions, the path made a U-turn in the hallway after which it entered the room they had just left. The purpose of these U-turns was to prevent the participants to predict the next room with certainty. A visit to one room is hereafter designated as a trial. During a CTX+ trial, a startle response occurred randomly between 8 and 10 s after entry, followed by a shock between 15 and 20 s after entry, and then a second startle response 24–25 s after entry, while on the way to leave the room. In order to increase the uncertainty of the CTX+ context, the second startle probe was replaced with a second shock on four visits. Thus, the total amount of shocks was 14 and the total amount of startle probes were 16. The CTX- trials mirrored the CTX + trial but did not deliver any shocks. During the time participants traversed the hallway, a total of 16 startle probes were also administered. The VR-session lasted 15 min, after which participants were asked to rate their discomfort and fear during the procedure. They were also asked about possible contingency awareness, and in that case to describe that contingency. Overall, Session 1 lasted about 45 min.

#### Session 2

In session 2, participants were randomly assigned to either the novel exploration task or the control task (a visual attention task). Participants assigned to the novel exploration task were informed that they were going to explore a walled-in park in virtual reality. To encourage exploration they were told that they were free to go wherever they wanted, and given the task to try to find three objects that were out of place in a park (a bathtub, a giant banana, and a giant apple). They were taught how to move using a standard hand-held game controller. The exploration lasted ten minutes during which participants movement were measured. After the exploration, participants were asked if they found the objects, and if they could place them on a printed map of the park, as well if they experienced any discomfort. Participants assigned to the visual attention task were placed in front of a computer and asked to follow the written instructions. The visual attention task was taken from the online experiment library of E-prime 3.0 (Psychology Software Tools, Pittsburgh, PA). During the task, the participant was asked to indicate the direction of the central arrow in three types of trials: congruent (e.g., <<<<< ), incongruent (e.g., >><>> ), and neutral (e.g., 00 < 00). During the trials, a fixation is present in the centre of the display, and arrows are presented either below or above the fixation. The reasoning was that this task would not involve processing of 3D-information, and not be a novel exploration. Both the novel exploration and the visual attention task took 10 min to complete. After this, participant filled out the STAI, GAD-7, and PHQ-9 questionnaires. Then, the participant waited until one hour had passed after starting their respective task (novel exploration, visual attention). During that time, they could rest or read, but not watch moving images or play games on their phones. One hour after they had started their respective task, participants went through an extinction session in VR. The extinction was identical to the acquisition, but no shocks were delivered in either context. After the extinction, participants again rated their discomfort and fear. In total, session 2 lasted about 90 min.

#### Session 3

In session 3, participants went through reinstatement and re-extinction to the contexts in VR. The procedure was identical to the extinction during session 2 but contained a single unsignaled shock in the corridor at the start of the session. Again, participants rated their discomfort and fear. In total, session 3, lasted about 30 min.

### Statistical analysis

Startle probe responses were quantified as the maximum EMG response 20–200 ms after the onset of the startle probe, subtracting a baseline measure of the mean EMG magnitude in a 500 ms period prior to the onset of the probe. These responses were then transformed to T-Scores (z-score × 10 + 50) in accordance with previous studies^18,19^.

Skin conductance responses (SCRs) to the startle probes were collected, making 16 values for each of the contexts (CTX+, CTX− and COR). This method has been successful in demonstrating context conditioning in a previous study^20^. Note that during acquisition, a startle probe sometimes followed an electric shock while the skin conductance response from the shock was still ongoing. Because of this design flaw, these trials were eliminated, and thus, the number of SCRs to startle responses used for each experimental phase were; acquisition – 10 trials, extinction − 16 trials, and reinstatement – 16 trials. SCRs to context transitions were also collected. The opening of the door between the corridor and CTX was used as stimulus onset, because this was the moment in which participants realized which context they were about to enter. Skin conductance responses were low-pass filtered with a 3 Hz cutoff frequency and a 4th order Butterworth filter, and analysed with a trough-to-peak procedure calculating the difference between the maximum and minimum values 1–5 s following stimulus onset using the neurokit2-toolbox^21^. Responses were then square root transformed, mean range corrected^22^ and arranged in bins (10-trial measures—start: trials 1–3, middle: trials 4–7, end: trials 8–10; 16-trial measures—start: trials 1–5, middle: trials 6–11, end: trials 12–16).

Results analysis followed the statistical analysis plan pre-registered at https://osf.io/4yjev/. In short, repeated measures ANOVAs with context (CTX+, CTX−) and trial bins (start, middle, end), as within-group variables and group (novel exploration, control) as between-group variable, were performed for the dependent variables (Startle responses, SCR to startle probes, and SCR for context transitions) for each experimental phase respectively. Where Mauchly’s test of sphericity was significance, Greenhouse-Geisser correction was used (G-G correction). Full ANOVAs can be found in the Supplementary materials. Follow-up t-tests were used to clarify ANOVA results. Sample size was determined based on earlier studies on context conditioning in virtual reality^12^. According to a G*Power^23^ sensitivity analysis regarding the critical comparison of a Stimulus × Group interaction at the start of reinstatement, the current sample size (*N* = 54, α = 0.05, power = 0.80) would be able to detect an effect size of Cohens f = 0.19, which is in between a small and medium effect.

## Results

The groups did not differ in age (NE: m = 26.66, CON m = 25.66; t(56) = − 0.53, *p* = 0.60), sex (NE: 16 female, 13 male, CON: 13 female, 16 male); χ^2^(1,58) = 0.62, *p* = 0.60), or shock strength (NE = 34.05, CON = 30.19; t(56) = − 1.38, *p* = 0.18). The groups did not differ in fear and discomfort ratings during the three sessions (Table 1). The participants that completed the novel exploration task found most of the objects we asked them to seek (3 objects = 24 participants, 2 objects = 4 participants, 1 object = 1 participant), demonstrating that participants did perform the exploration task.

**Table 1:** Fear and discomfort ratings during the three sessions.

	CON	NE	t	*p*	Cohens d
Fear Session 1	14.24	13.17	0.80	0.78	0.073
Fear Session 2	11.41	6.10	1.60	0.12	0.43
Fear Session 3	6.67	5.56	− 0.39	0.70	− 0.11
Discomfort Session 1	34.72	28.07	1.32	0.19	0.35
Discomfort Session 2	16.31	9.79	1.60	0.12	0.44
Discomfort Session 3	10.93	8.07	1.00	0.32	0.27

*NE* Novel exploration group, *CON* Control group.

### Psychophysiology

Results from the three different outcome variables (startle responses, SCRs to startle responses, and SCRs to context transitions) were analysed separately and are presented individually below.

### Startle responses

#### Acquisition

**Fig. 2:**
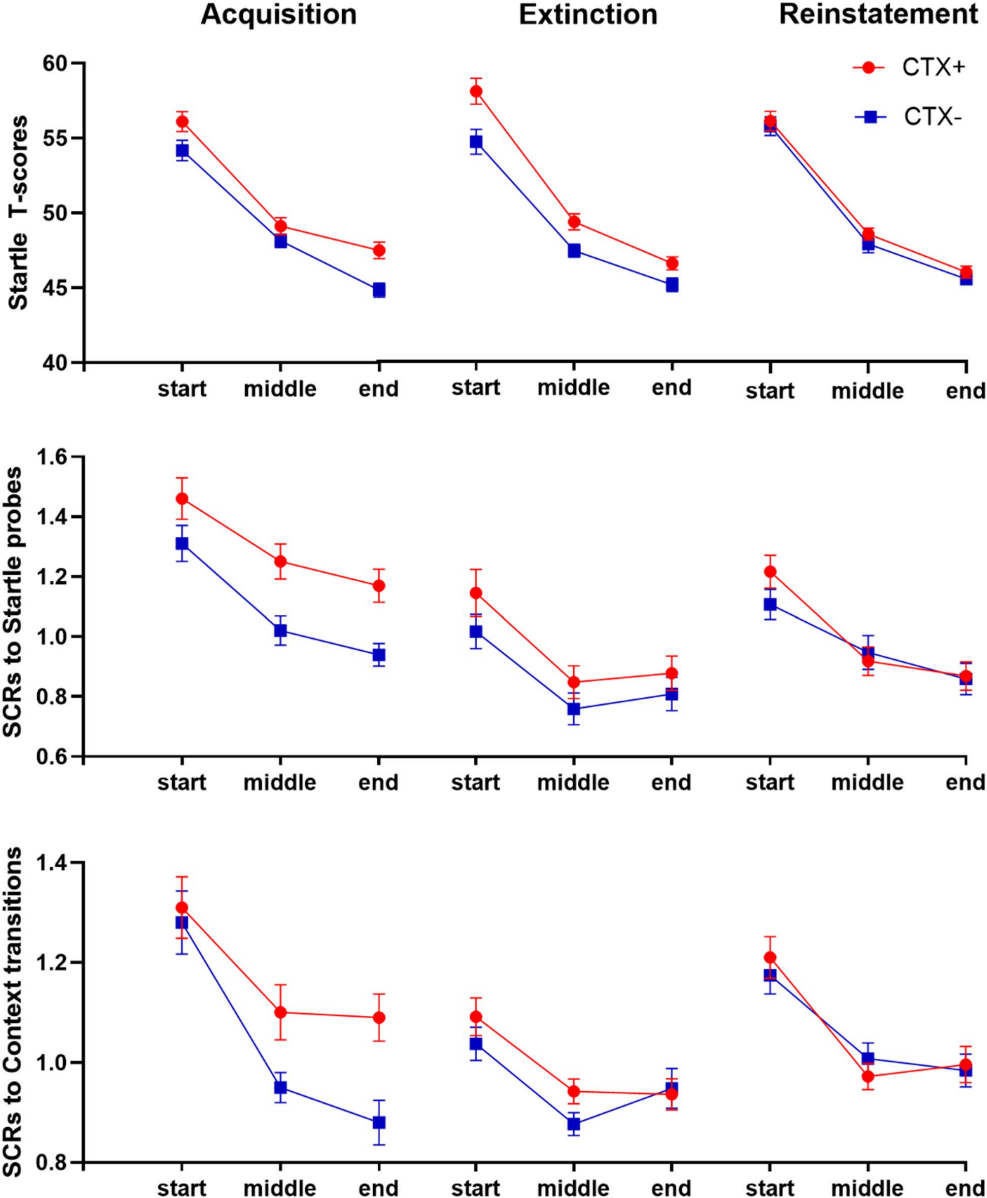
Summary of psychophysiology results. Top panel: Startle responses (T-scores) in CTX + and CTX− during acquisition, extinction, and reinstatement. Middle panel: Root-transformed mean range-corrected SCR to startle responses in CTX+ and CTX− during acquisition, extinction, and reinstatement. Bottom panel: Root-transformed mean range-corrected SCR to context transitions in CTX+ and CTX− during acquisition, extinction, and reinstatement.

A 2 × 3 × 2 rmANOVA using Context (CTX+, CTX−) and Trial (start, middle, end) as within-group variables, and Group (novel exploration, control) as a between-group variable, showed evidence of context conditioning through a main effect of Context, F(1,52) = 9.18, *p* < 0.01, η^2^ = 0.15. There was also a main effect of Trial, F(2,104) = 138.06, *p* < 0.001, η^2^ = 0.73, but no other main or interaction effects were observed (Fig. 2 top panel, Supplementary Tables 1 and 2). Thus, startle responses indicated that contextual fear learning took place and did not differ between the experimental groups.

#### Extinction

A 2 × 3 × 2 rmANOVA using Context (CTX+, CTX−), Trial (start, middle, end) as within-group variables and Group (novel exploration, control) as a between-group variable, showed a main effect of Context, F(1,47) = 10.04, *p* = 0.003, η^2^ = 0.18, and Trial, F(1.60,75.28) = 138.69, *p* < 0.001 (G-G corrected), η^2^ = 0.75. The Context ×Trial interaction, often used as a measure of extinction, did not reach significance, F(1.54,72.21) = 1.91, *p* = 0.17 (G-G corrected), η^2^ = 0.04 (Fig. 2 top panel, Supplementary Tables 1 and 3). Exploratory t-tests showed that startle responses showed a difference between CTX + and CTX− at start of extinction; start, t(48) = 2.59, *p* = 0.01, which did not fade much to the middle, t(48) = 2.52, *p* = 0.02, or end, t(48) = 2.52, *p* = 0.02, which was unexpected. Thus, startle responses showed weak evidence for extinction learning, suggesting that the extinction session may have been too short, but see results on SCRs.

#### Reinstatement

A 2 × 3 × 2 rmANOVA using Context (CTX+, CTX−), trial (start, middle, end) as within-group variables and Group (novel exploration, control) as between-group variable, showed a main effect of trial, F(1.96,94.09) = 191,753, *p* < 0.001, η^2^ = 0.80, but no other effects were observed. (Fig. 2 top panel, Supplementary Tables 1 and 4).

An analysis comparing the change from the end of extinction to the start of reinstatement, using a 2 × 2 rmANOVA with Context (CTX+, CTX−), trial (end of extinction, start of reinstatement) as within-group variables and Group (novel exploration, control) as between-group variable was run on participants who had non-corrupted EMG data for both session 2 and 3. Results showed a main effect of Trial, F(1,44) = 371.257, *p* < 0.001, η^2^ = 0.89, reflecting a general increase in responses from end of extinction to start of reinstatement. There were also a Context ×Trial, F(1,44) = 5.321, *p* = 0.03, η^2^ = 0.11, and a Context ×Trial × Group effect, F(1, 44) = 5.565. *p* = 0.02, η^2^ = 0.11 (Supplementary Table 5). The Context ×Trial effect reflected a shift from higher responses in CTX + as compared to CTX− at the end of extinction, t(45) = 2.64, *p* = 0.01, d = 0.39, to no differentiation between contexts at start of reinstatement, t(45) = − 1.01, *p* = 0.32, d = − 0.15. The Context ×Trial × Group effect reflected that the control group displayed non-typical conditioning data at start of reinstatement consisting of higher responses to CTX− than CTX+, t(22) = −2.27, *p* = 0.03, d = −0.47, whereas the novel exploration group did not differ in their startle responses to CTX + and CTX−, t(22) = 0.56, *p* = 0.58, d = 0.12. Thus, no reinstatement of fear learning could be detected. A Bayesian t-test comparing differential conditioning (CTX+ - CTX−) between the groups was in favour for the null hypothesis both at start of reinstatement, BF_01_ = 2.51, and over the whole reinstatement session, BF_01_ = 2.03.

### Skin conductance responses to startle probes

#### Acquisition

A 2 × 3 × 2 rmANOVA using Context (CTX+, CTX−) and Trial (start, middle, end) as within-group variables and Group (novel exploration, control) as a between-group variable, showed a main effect of Context, F(1,56) = 27.76, *p* < 0.001, η^2^ = 0.33, and Trial, F(2,112) = 24.51, *p* < 0.001, η^2^ = 0.30, but no other effects (Fig. 2, middle panel, supplementary Tables 6 and 7). Thus, SCRs to startle probes showed a conditioning effect, indicating fear learning that did not differ between experimental groups.

#### Extinction

A 2 × 3 × 2 rmANOVA using Context (CTX+, CTX−) and Trial (start, middle, end) as within-group variables and Group (novel exploration, control) as a between-group variable, showed main effects of Context, F(1,53) = 6.454, *p* = 0.014, η^2^ = 0.11, and Trial, F(1.67,88.21) = 17.28, *p* < 0.001, η^2^ = 0.25. The Context ×Trial effect was not significant, F(2,106) = 0.81, *p* = 0.45, η^2^ = 0.02, but exploratory t-tests showed that responses to CTX + and CTX- differed during start, t(54) = 2.11, *p* = 0.04, d = 0.29, and middle, t(54) = 2.25, *p* = 0.03, d = 0.30, of extinction, but not during the end, t(54) = 1.56, *p* = 0.13, d = 0.21, suggesting that extinction were taking place, but a bit too slow to be caught during our 10 trials. No other effects were observed (Fig. 2 middle panel, Supplementary Tables 6 and 8).

#### Reinstatement

A 2 × 3 × 2 rmANOVA using Context (CTX+, CTX−), Trial (start, middle, end) as within-group variables and group (novel exploration, control) as between-group variable, showed a main effect of Trial, F(1.68,87.45) = 18,49, *p* < 0.001 G-G corrected), η^2^ = 0.26. The Context ×Trial effect was not significant, F(2,104) = 2.16, *p* = 0.12, η^2^ = 0.04, but exploratory t-tests showed that participants differed between CTX+ and CTX− at start of reinstatement, t(53) = 2.03, *p* < 0.05, but not at middle, t(53) = − 0.748, *p* = 0.46, or end, t(54) = 0.14, *p* = 0.89, suggesting that contextual fear was reinstated, and later extinguished. Because there were no effects of Group, it appears the novel exploration had no impact on the SCRs to startle probes during reinstatement (Fig. 2 middle panel, Supplementary Tables 6 and 9). Analysis comparing the change from end of extinction to start of reinstatement also did not find any Group effects (Supplementary Table 10). A Bayesian t-test comparing differential conditioning between the groups was in favour for the null hypothesis both at start of reinstatement, BF_01_ = 3.58, and over the whole reinstatement session, BF_01_ = 3.64.

### Skin conductance response to context transitions

#### Acquisition

A 2 × 3 × 2 rmANOVA using Context (CTX+, CTX−), Trial (start, middle, end) as within-group variables and Group (novel exploration, control) as a between-group variable, showed a main effect of Context, F(1,56) = 21.21, *p* < 0.001, η^2^ = 0.28, and Trial, F(1.50,84.08) = 17.97, *p* < 0.001 G-G corrected, η^2^ = 0.24, and a near significant Context ×Trial interaction, F(2,112) = 2.51, *p* = 0.09, η^2^ = 0.04 (Fig. 2 lower panel, Supplementary Tables 11 and 12). Thus, SCRs to context transitions showed a conditioning effect, but no differences between groups, indicating fear learning did not differ between groups.

#### Extinction

A 2 × 3 × 2 rmANOVA using Context (CTX+, CTX−), Trial (start, middle, end) as within-group variables and Group (novel exploration, control) as a between-group variable showed an effect of Trial, F(1.47,77.63) = 9.45, *p* < 0.001 G-G corrected, η^2^ = 0.15 and a near-significant effect of Context, F(1,53) = 3.51, *p* = 0.07. The context by trial effect was not significant, F(2,106) = 1.85, *p* = 0.16, but exploratory t-tests showed that participants tended to separate between CTX + and CTX− at start, t(58) = 1.91, *p* = 0.06, d = 0.26, and middle, t(58) = 2.52, *p* = 0.01, d = 0.34, of extinction, but not at the end, t(58) = − 0.32, *p* = 0.75, d = − 0.04, suggesting extinction took place (Fig. 2 lower panel, Supplementary Tables 11 and 13).

#### Reinstatement

The 2 × 3 × 2 rmANOVA using Context (CTX+, CTX−), Trial (start, middle, end) as within-group variables and Group (novel exploration, control) as a between-group variable only showed a main effect of Trial, F(1.52,78.98) = 21.14, *p* < 0.001 G-G corrected, η^2^ = 0.29. (Fig. 2 lower panel, Supplementary Tables 11 and 14). Analysis comparing the change from end of extinction to start of reinstatement also did not find any Group effects (Supplementary Table 15). For context transitions, no reinstatement of fear learning could be detected at start of reinstatement, t(53) = − 0.13, *p* = 0.90. A Bayesian t-test comparing differential conditioning between the groups was in favour for the null hypothesis both at start of reinstatement, BF_01_ = 3.35, and over the whole reinstatement session, BF_01_ = 3.15.

### Contingency awareness

After the acquisition session, participants were asked if they had discovered some sort of pattern regarding when they received the shocks. If they reported that they had, they were asked to describe that pattern. Participants were good at detecting that there was a greater chance of shocks in one room compared to the other, but few were 100% sure. Many reported that ”there were more shocks in one room”, or gave a probability (e.g. 70/30 between the rooms), allowing for a chance that there were shocks also in the other room. When classifying all answers that clearly stated that the CTX + room was riskier than the CTX− room as contingency awareness, 43 out of 58 (74%; novel exploration *N* = 21, control *N* = 22) showed contingency awareness, and 15 out of 58 (26%; novel exploration *N* = 8, control *N* = 7) did not. Using this classification, we tested whether groups differed in contingency awareness using a χ^2^-test, which did not show significance: χ^2^(1) = 0.35, *p* = 0.56. Some participants also had more complex theories of how the shocks were connected to specific visual details, or specific head movements. Hence, in spite of the very simple contingency of 100% reinforcement in one context, this contingency was not obvious for some of the participants.

## Discussion

The current study examined whether a novel exploration task, performed shortly before extinction learning of contextual fear, could affect remaining contextual fear 24 h later, in line with the behavioural tagging hypothesis. A second aim of the study was to confirm that a virtual reality contextual conditioning procedure can instil contextual fear, measured by SCRs and startle probes, without the use of subjective ratings during the procedure, or the commonly used semi-instructed procedure where participants are instructed to pay attention to and discover an association between stimuli^12^.

The study could not find any evidence that the novel exploration procedure performed day two affected fear responses during reinstatement day three. It is unclear to us why the control group displayed larger startle responses to CTX− as compared to CTX + at start of reinstatement, as this group only performed a visual control task. In addition, both SCR measures did not show this pattern, suggesting that if the visual control task somehow influenced the conditioning processes, it only affected startle responses.

We conclude that the study did not manage to translate, or conceptually replicate, the previous findings on behavioural tagging in rodents^11^. There may be many reasons for this, and we will consider a few: The basic process of learning that a context is unsafe through repeated pairings of a context and an electric shock is similar for rodents and humans, and presumably rests upon similar neural functions. However, there are differences between context conditioning in humans and rodents that may hinder translation of results from rodents to humans. For instance, human context conditioning includes two contexts, as a control context (CTX−) is needed to contrast the conditioning context (CTX+). This may affect the learning and memory formation, as the resulting conditioned response (CTX+ to CTX−) is dependent on the response to both an unsafe and a safe environment. In addition, unlike rodents, humans are prone to cognition around the conditioning process. Although we tried to limit the influence of cognitive processes by excluding subjective fear ratings and instructions about contingency, several participants told us of how they expected jump scares towards the end of the procedures, and how they considered if distinct parts of the rooms were more unsafe. This cognition may also be a hindrance towards translational studies in context conditioning. Moreover, a novel exploration task for a rodent is exposure to a novel open field. We tried to emulate this by having participants traverse a park in virtual reality. However, for rodents, exploring an open field constitutes a stressful situation, where it is exposed to danger^14^, whereas humans are not particularly stressed by open fields. In humans, perhaps enclosed spaces would enhance fear responses, as they have limited escape routes. In any case, it is likely that the emotional impact of an open field is very different for rodents and humans.

It is possible that the VR exploration task was not considered very novel by the participants. It has been suggested that the impact of novelty on memory is mostly dependent on the unexpectedness of stimuli^24^. In mimicking the use of the rodent open field test, we ended up with a procedure where stimuli (the virtual garden) were not very surprising, and the novelty, for participants unfamiliar with VR, may have been limited to the use of VR itself. On the other hand, it is possible that the VR context conditioning task, in addition to the VR exploration task, was considered a novel experience by the participants. This could possibly contribute to the extinction memory strength across groups, attenuating the return of fear during reinstatement, occluding group differences. Another possibility is that the visual attention task used as control condition was somehow considered to be as novel as our novel exploration task, leading to comparable extinction memory strength in the two groups. We would not expect this, because a prerequisite for a task to affect a memory encoding through behavioural tagging mechanisms appears to be that it recruits the same neural structures^1^, and while both novel exploration and context conditioning are assumed to depend on spatial processing and the hippocampus, a visual attention task is not. Still, it remains a possibility.

Regarding the secondary aim, the study showed that a virtual reality procedure, where participants receive electric shocks in one context (CTX+), but not another (CTX−), produced context fear conditioning all three outcome measures, also without a semi-instructed procedure or subjective ratings during the procedure. This may be important for the possibility of translating results from rodents to humans, as no such instructions or ratings, or the cognitive processes they trigger, can be found in rodents.

Some evidence of extinction was noted in both SCRs to context transitions and SCR to startle probes, but startle responses showed weak evidence for extinction. Thus, our data suggests that SCRs may be more sensitive to extinction than startle responses, but no firm conclusion can be drawn. SCRs to startle probes displayed a return of fear on day three, which could not be noted with either startle probes or SCRs to context transitions. Alternatively, there was a generalized reinstatement, leading to increased responses in both CTX + and CTX−. This is in line with data, which increased from end of extinction to start of reinstatement in all three measures.

In summary, the three measures differed somewhat in capturing the processes associated with the experimental setup for fear memory research. Future research and meta-analyses must decide whether the three measures differ in their usefulness to measure different context conditioning processes, or whether these results are stemming from power problems, and that the results of the different measures would converge if power were to be significantly increased.

### Future directions

Results supporting the behavioural tagging hypothesis have been shown for a variety of hippocampal-dependent tasks^1^. If a novel exploration task is unsuitable for producing the effect in humans, perhaps in future studies, a better manipulation would be a task that simply engages the hippocampus as much as possible, for example a labyrinth navigation task. Because context conditioning is dependent on hippocampal activity^10^, it remains an interesting tool to produce these effects in humans. Indeed, as the present study shows, contextual fear can be installed in humans even without experimentally induced cognitive processes from instructions or subjective ratings, further strengthening the case of using it as a tool for translating behavioural tagging results from animals to humans.

## Conclusion

A novel exploration shortly before fear extinction was not found to have any effect on the fear responses during fear reinstatement 24 h later. Thus, this study did not manage to translate the rodent results to humans. Future research could test other manipulations that promotes hippocampal activity, such as navigation tasks, and perhaps continue to develop the context conditioning task to improve the strength of the conditioning effect.

## Electronic supplementary material

Below is the link to the electronic supplementary material.

Supplementary Material 1

## Data Availability

Data are available in the Open Science Framework: https://osf.io/4yjev/.
