# Supplementary material for: No effect of novel exploration on the consolidation of extinction learning in human context conditioning
Source: Sci Rep. 2025 Jun 20;15:20151. doi: 10.1038/s41598-025-05235-2 (PMC12181346; doi:10.1038/s41598-025-05235-2)
Supplement: Supplementary file 1 — Supplementary Material 1 [file 41598_2025_5235_MOESM1_ESM.pdf]

Supplementary materials

No Effect of Novel Exploration on the Consolidation of Extinction Learning in Human Context Conditioning

Thomas Agren[1,2], Johannes Björkstrand[3], Jörgen Rosén[4].

[1]*Department of Psychology, Uppsala University, Uppsala, Sweden*
[2]*Department of Occuptional Health, Psychology, and Sports Science,University of Gävle, Gävle, Sweden*
[3]*Department of Psychology, Lunds University, Lund, Sweden*
[4]*Department of Clinical Neuroscience, Karolinska Institutet, Stockholm, Sweden*

Open Science Framework: https://osf.io/4yjev/

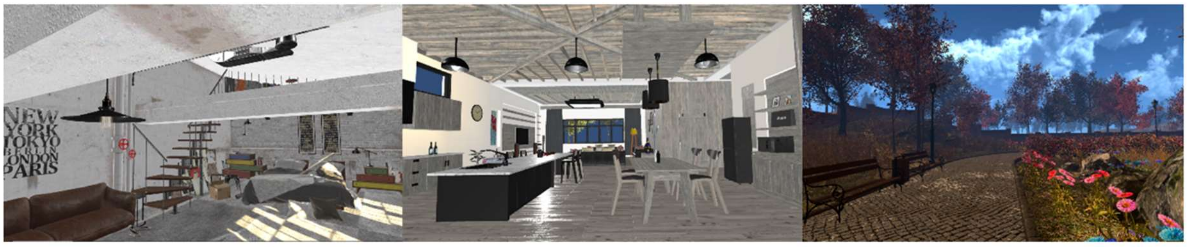

Supplementary Figure S1. Examples of the virtual environments. Left and middle panel depicts the two rooms that served as CTX+ and CTX-. The right panel is a screenshot from the park in which the novel exploration took place.

Supplementary table 1. Startle responses (T-scores) in CTX+ and CTX- across all experimental procedures.

	Trial bin[1]					
	Start		Mid		End	
	CTX+	CTX-	CTX+	CTX-	CTX+	CTX-
**All participants**						
Acquisition	56.11 (.67)	54.17 (.68)	49.12 (.58)	48.15 (.46)	47.50 (.56)	44.86 (.47)
Extinction	58.11 (.86)	54.73 (.84)	49.36 (.53)	47.42 (.43)	46.57 (.43)	45.14 (.45)
Reinstatement	56.11 (.65)	55.76 (.61)	48.52 (.40)	47.86 (.58)	45.98 (.41)	45.50 (.39)
**Novel exploration**						
Acquisition	56.10 (.98)	53.59 (.80)	49.26 (.74)	48.01 (.63)	47.54 (.91)	45.55 (.79)
Extinction	58.45 (1.4)	53.88 (1.2)	49.33 (.62)	48.16 (.70)	46.65 (.61)	45.61 (.54)
Reinstatement	56.44 (.88)	55.71 (.78)	49.12 (.60)	47.43 (.80)	45.29 (.65)	45.70 (.61)
**Visual control**						
Acquisition	56.11 (.92)	54.70 (1.1)	49.00 (.89)	48.23 (.67)	47.47 (.69)	44.21 (.51)
Extinction	57.78 (1.00)	55.56 (1.2)	49.38 (.86)	47.72 (.49)	46.48 (.63)	44.70 (.71)
Reinstatement	55.74 (.97)	57.90 (.92)	47.86 (.50)	48.33 (.86)	45.65 (.47)	45.28 (.47)

Note. Standard error is displayed in parenthesis. [1] Trials within each experimental procedure were partitioned into three bins (start, mid, end; trials 1–5, 6–11, 12–16.

Supplementary table S2. ANOVA for context conditioning day 1 as measured by startle response (T-scores).

	SS	*df*	*MS*	*F*	*p*	η_p^2
Context	278.425	1.000	278.425	9.177	0.004	0.150
Context × Group	0.278	1.000	0.278	0.009	0.924	<0.001
Error (Context)	1577.620	52	30.339			
Trial	4599.109	2	2299.555	138.063	<0.001	0.73
Trial × Group	21.899	2	10.950	0.657	0.520	0.012
Error (Trial)	1732.212	104	16.656			
Context × Trial	36.588	1.776	20.602	1.059	0.344	0.020
Context × Trial × Group	20.778	1.776	11.700	0.602	0.531	0.011
Error (Context × Trial)	1795.722	92.347	19.445			
Between-subjects effects						
Group	215.095	1	215.095	0.133	0.717	0.003
Error	84382.552	52	1622.741			

Note. Results form 2 × 3 × 2 analyses of variance of startle probes with Context (CTX+, CTX-) and Trial (start, middle, end) as within-subject variables and Group (novel exploration, control) as between-subjects variable. Greenhouse-Geisser correction is applied where needed.

Supplementary table S3. ANOVA for extinction day 2 as measured by startle response (T-scores).

	SS	*df*	*MS*	*F*	*p*	η_p^2
Context	370.530	1.000	370.530	10.042	0.003	0.176
Context × Group	0.026	1.000	0.026	0.001	0.979	<0.001
Error (Context)	1734.144	47	36.897			
Trial	5950.677	1.602	3715.234	138.687	<0.001	0.747
Trial × Group	21.019	1.602	13.123	0.490	0.573	0.010
Error (Trial)	2016.641	75.280	26.789			
Context × Trial	52.263	1.536	34.016	1.905	0.165	0.039
Context × Trial × Group	50.466	1.536	32.847	1.839	0.174	0.038
Error (Context × Trial)	1289.472	72.211	17.857			
Between-subjects effects						
Group	4.311	1	4.311	0.695	0.409	0.015
Error	291.342	47	6.199			

Note. Results form 2 × 3 × 2 analyses of variance of startle probes with Context (CTX+, CTX-) and Trial (start, middle, end) as within-subject variables and Group (novel exploration, control) as between-subjects variable. Greenhouse-Geisser correction is applied where needed.

Supplementary table 4. ANOVA for reinstatement day 3 as measured by startle response (T-scores).

	SS	*df*	*MS*	*F*	*p*	η_p^2
Context	1.163	1.000	1.163	0.059	0.81	0.001
Context × Group	57.856	1.000	57.856	2.950	0.092	0.058
Error (Context)	941.315	48	19.611			
Trial	6295.004	1.960	3211.338	191.725	<0.001	0.800
Trial × Group	21.371	1.960	10.902	0.651	0.524	0.013
Error (Trial)	1576.009	94.092	16.750			
Context × Trial	26.378	1.715	15.383	+-968	0.373	0.020
Context × Trial × Group	23.736	1.715	13.843	0.871	0.408	0.018
Error (Context × Trial)	1307.927	82.306	15.891			
Between-subjects effects						
Group	0.011	1	0.011	0.074	0.787	0.002
Error	6.840	48	0.143			

Note. Results form 2 × 3 × 2 analyses of variance of startle probes with Context (CTX+, CTX-) and Trial (start, middle, end) as within-subject variables and Group (novel exploration, control) as between-subjects variable. Greenhouse-Geisser correction is applied where needed.

Supplementary table S5. ANOVA exploring the change from the end of extinction to the start of reinstatement in startle response (T-scores).

	SS	*df*	*MS*	*F*	*p*	η_p^2
Context	2.287	1.000	2.287	0.130	0.720	0.003
Context × Group	18.311	1.000	18.311	1.042	0.313	0.023
Error (Context)	773.198	44	17.573			
Trial	5110.880	1	5110.880	371.257	<0.001	0.894
Trial × Group	18.454	1	18.454	1.340	0.253	0.030
Error (trial)	605.723	44	13.766			
Contex × Trial	76.574	1	76.574	5.321	0.026	0.108
Context × Trial × Group	80.092	1	80.092	5.565	0.023	0.112
Error (Context × Trial)	633.235	44	14.329			
Between-subjects effects						
Group	0.969	1.00	0.969	0.086	0.771	0.002
Error	497.916	44	11.316			

Note. Results form 2 × 2 × 2 analyses of variance of startle probes with Stimulus (CS+, CS) and Trial (end of extinction, start of reinstatement) as within-subject variables and Group (novel exploration, control) as between-subjects variable.

Supplementary table 6. SCR to startle probes in CTX+ and CTX- across all experimental procedures.

	Trial bin[1]					
	Start		Mid		End	
	CTX+	CTX-	CTX+	CTX-	CTX+	CTX-
**All participants**						
Acquisition	1.46 (.07)	1.31 (.06)	1.25 (.06)	1.02 (.05)	1.17 (.06)	0.94 (.04)
Extinction	1.15 (.07)	1.02 (.05)	0.85 (.05)	0.76 (.04)	0.88 (.04)	0.81 (.05)
Reinstatement	1.22 (.06)	1.11 (.05)	0.92 (.05)	0.95 (.06)	0.87 (.05)	0.86 (.05)
**Novel exploration**						
Acquisition	1.57 (.10)	1.39 (.10)	1.29 (.09)	1.07 (.08)	1.21 (.08)	0.95 (.05)
Extinction	1.14 (.09)	1.02 (.07)	0.81 (.05)	0.73 (.05)	0.90 (.06)	0.79 (.07)
Reinstatement	1.22 (.08)	1.10 (.08)	0.92 (.07)	0.94 (.07)	0.85 (.07)	0.87 (.08)
**Visual control**						
Acquisition	1.35 (.09)	1.23 (.06)	1.22 (.07)	0.98 (.05)	1.13 (.07)	0.94 (.06)
Extinction	1.17 (.10)	1.02 (.08)	0.89 (.08)	0.80 (.07)	0.85 (.07)	0.84 (.07)
Reinstatement	1.22 (.08)	1.12 (.06)	0.93 (.07)	0.97 (.09)	0.88 (.06)	0.85 (.07)

Note. Standard error is displayed in parenthesis. [1] Trials within each experimental procedure were partitioned into three bins (start, mid, end; trials 1–5, 6–11, 12–16. Skin conductance responses were root-transformed and mean range corrected over all experimental procedures.

Supplementary table 7. ANOVA for context conditioning day 1 as measured by SCRs to startle probes.

	SS	*df*	*MS*	*F*	*p*	η_p^2
Context	3.572	1.000	3.572	27.764	<0.001	0.331
Context × Group	0.020	1.000	0.020	0.159	0.692	0.003
Error (Context)	7.204	56	0.129			
Trial	6.8250	1.608	4.243	24.508	<0.001	0.304
Trial × Group	0.332	1.608	0.206	1.192	0.307	0.021
Error (Trial)	15.594	90.069	0.173			
Context × Trial	0.126	2	0.063	0.724	0.487	0.013
Context × Trial × Group	0.031	2	0.016	0.180	0.836	0.003
Error (Context × Trial)	9.720	112	0.087			
Between-subjects effects						
Group	0.950	1	0.950	1.857	0.178	0.032
Error	28.639	56	0.511			

Note. Results form 2 × 3 × 2 analyses of variance of startle probes with Context (CTX+, CTX-) and Trial (start, middle, end) as within-subject variables and Group (novel exploration, control) as between-subjects variable. SCRs are root transformed and mean range corrected- Greenhouse-Geisser correction is applied where needed. SCRs are root transformed and mean range corrected

Supplementary table S8. ANOVA for extinction day 2 as measured by SCRs to startle probes.

	SS	*df*	*MS*	*F*	*p*	η_p^2
Context	0.734	1.000	0.734	6.454	0.014	0.109
Context × Group	0.008	1.000	0.008	0.070	0.792	0.001
Error (Context)	6.024	53	0.114			
Trial	5.101	1.664	3.065	17.279	<0.001	0.248
Trial × Group	0.103	1.664	0.062	0.348	0.668	0.007
Error (Trial)	15.645	88.207	0.177			
Context × Trial	0.068	2	0.034	0.808	0.449	0.015
Context × Trial × Group	0.069	2	0.035	0.818	0.444	0.015
Error (Context × Trial)	4.483	106	0.042			
Between-subjects effects						
Group	0.081	1	0.081	0.224	0.638	0.004
Error	19.200	53	0.362			

Note. Results form 2 × 3 × 2 analyses of variance of startle probes with Context (CTX+, CTX-) and Trial (start, middle, end) as within-subject variables and Group (novel exploration, control) as between-subjects variable. Greenhouse-Geisser correction is applied where needed. SCRs are root transformed and mean range corrected

Supplementary table 9. ANOVA for reinstatement day 3 as measured by SCRs startle probes.

	SS	*df*	*MS*	*F*	*p*	η_p^2
Context	0.068	1.000	0.068	1.123	0.294	0.021
Context × Group	0	1.000	0	0.008	0.929	<0.001
Error (Context)	3.154	52	0.061			
Trial	5.311	1.682	3.157	18.486	<0.001	0.262
Trial × Group	0.001	1.682	0.001	0.004	0.99	<0.001
Error (Trial)	14.941	87.478	0.171			
Context × Trial	0.298	2	0.149	2.160	0.120	0.040
Context × Trial × Group	0.022	2	0.011	0.159	0.853	0.003
Error (Context × Trial)	7.165	104	0.069			
Between-subjects effects						
Group	0.017	1	0.017	0.040	0.841	0.001
Error	21.601	52	0.415			

Note. Results form 2 × 3 × 2 analyses of variance of startle probes with Context (CTX+, CTX-) and Trial (start, middle, end) as within-subject variables and Group (novel exploration, control) as between-subjects variable. Greenhouse-Geisser correction is applied where needed. SCRs are root transformed and mean range corrected

Supplementary table 10. ANOVA exploring the change from the end of extinction to the start of reinstatement in skin conductance responses to startle probes.

	SS	*df*	*MS*	*F*	*p*	η_p^2
Context	0.412	1.000	0.412	6.590	0.013	0.112
Stimulus × Group	0.050	1.000	0.050	0.795	0.377	0.015
Error (stimulus)	3.249	52	0.062			
Trial	5.441	1	5.441	16.491	<0.001	0.241
Trial × Group	0.004	1	0.004	0.013	0.908	<0.001
Error (trial)	17.157	52	0.330			
Stimulus × Trial	0.032	1	0.032	0.475	0.494	0.009
Stimulus × Trial × Group	0.021	1	0.021	0.311	0.580	0.006
Error (Stimulus × Trial)	3.541	52	0.068			
Between-subjects effects						
Group	0.001	1.00	0.001	0.014	0.906	<0.001
Error	4.655	52	0.090			

Note. Results form 2 × 2 × 2 analyses of variance of skin conductance responses to startle probes with Stimulus (CS+, CS) and Trial (end of extinction, start of reinstatement) as within-subject variables and Group (novel exploration, control) as between-subjects variable. SCRs are root transformed and mean range corrected

Supplementary table 11. Mean skin conductance responses to context transitions into CTX+ and CTX- across all experimental procedures.

	Trial bin[1]					
	Start		Mid		End	
	CTX+	CTX-	CTX+	CTX-	CTX+	CTX-
**All participants**						
Acquisition	1.31 (.06)	1.28 (.06)	1.10 (.05)	0.95 (.03)	1.09 (.05)	0.88 (.04)
Extinction	1.09 (.06)	1.00 (.06)	0.84 (.04)	0.73 (.04)	0.83 (.05)	0.85 (.07)
Reinstatement	1.29 (.07)	1.23 (.06)	0.89 (.04)	0.95 (.05)	0.93 (.06)	0.91 (.06)
**Novel exploration**						
Acquisition	1.39 (.10)	1.30 (.10)	1.09 (.08)	0.91 (.04)	1.11 (.07)	0.90 (.07)
Extinction	1.08 (.08)	0.94 (.07)	0.81 (.06)	0.70 (.05)	0.84 (.07)	0.83 (.11)
Reinstatement	1.31 (.10)	1.29 (.10)	0.89 (.07)	0.93 (.08)	1.00 (.09)	0.89 (.07)
**Visual control**						
Acquisition	1.24 (.08)	1.26 (.08)	1.11 (.08)	0.99 (.05)	1.07 (.07)	0.86 (.05)
Extinction	1.09 (.10)	1.06 (.08)	0.87 (.06)	0.77 (.06)	0.83 (.08)	0.87 (.08)
Reinstatement	1.27 (.10)	1.18 (.07)	0.88 (.08)	0.95 (.07)	0.86 (.08)	0.93 (.09)

Note. Standard error is displayed in parenthesis. [1] Trials within each experimental procedure were partitioned into three bins (start, mid, end; trials 1–3, 4–7, 8–10. Skin conductance responses were root-transformed and mean range corrected.

Supplementary table 12. ANOVA for context conditioning day 1 as measured by SCRs to context transitions.

	SS	*df*	*MS*	*F*	*p*	η_p^2
Context	1.559	1.000	1.559	21.206	<0.001	0.275
Context × Group	0.062	1.000	0.062	0.841	0.363	0.015
Error (Context)	4.116	56	0.073			
Trial	6.636	1.501	4.420	17.966	<0.001	0.243
Trial × Group	0.343	1.501	0.228	0.928	0.375	0.016
Error (Trial)	20.685	84.075	0.246			
Context × Trial	0.480	2	0.240	2.514	0.086	0.043
Context × Trial × Group	0.049	2	0.024	0.254	0.776	0.005
Error (Context × Trial)	10.702	112	0.096			
Between-subjects effects						
Group	0.060	1	0.060	0.204	0.654	0.004
	16.508	56	0.295			

Note. Results form 2 × 3 × 2 analyses of variance of startle probes with Context (CTX+, CTX-) and Trial (start, middle, end) as within-subject variables and Group (novel exploration, control) as between-subjects variable. Greenhouse-Geisser correction is applied where needed. SCRs are root transformed and mean range corrected

Supplementary table 13. ANOVA for extinction day 2 as measured by SCRs to context transitions.

	SS	*df*	*MS*	*F*	*p*	η_p^2
Context	0.276	1.000	0.276	3.514	0.066	0.062
Context × Group	0.057	1.000	0.057	0.720	0.400	0.013
Error (Context)	4.160	53	0.078			
Trial	3.978	1.465	2.716	9.448	<0.001	0.151
Trial × Group	0.051	1.465	0.035	0.121	0.822	0.002
Error (Trial)	22.318	77.633	0.287			
Context × Trial	0.259	2	0.130	1.852	0.162	0.034
Context × Trial × Group	0.039	2	0.022	0.281	0.755	0.005
Error (Context × Trial)	7.417	106	0.070			
Between-subjects effects						
Group	0.205	1	0.205	0.596	0.444	0.011
Error	18.217	53	0.344			

Note. Results form 2 × 3 × 2 analyses of variance of startle probes with Context (CTX+, CTX-) and Trial (start, middle, end) as within-subject variables and Group (novel exploration, control) as between-subjects variable. Greenhouse-Geisser correction is applied where needed. SCRs are root transformed and mean range corrected

Supplementary table 14. ANOVA for reinstatement day 3 as measured by SCRs context transitions.

	SS	*df*	*MS*	*F*	*p*	η_p^2
Context	0.003	1.000	0.003	0.025	0.874	<0.001
Context × Group	0.042	1.000	0.042	0.338	0.563	0.006
Error (Context)	6.385	52	0.123			
Trial	8.391	1.519	5.525	21.142	<0.001	0.289
Trial × Group	0.088	1.519	0.058	0.222	0.739	0.004
Error (Trial)	20.638	78.979	0.261			
Context × Trial	0.185	1.704	0.109	0.890	0.400	0.017
Context × Trial × Group	0.210	1.704	0.124	1.010	0.358	0.019
Error (Context × Trial)	10.832	88.601	0.122			
Between-subjects effects						
Group	0.142	1	0.142	0.357	0.553	0.007
Error	20.658	52	0.397			

Note. Results form 2 × 3 × 2 analyses of variance of startle probes with Context (CTX+, CTX-) and Trial (start, middle, end) as within-subject variables and Group (novel exploration, control) as between-subjects variable. Greenhouse-Geisser correction is applied where needed. SCRs are root transformed and mean range corrected

Supplementary table 15. ANOVA exploring the change from the end of extinction to the start of reinstatement in skin conductance responses to context transitions.

	SS	*df*	*MS*	*F*	*p*	η_p^2
Context	0.023	1	0.023	0.167	0.684	0.003
Context × Group	0.001	1	0.001	0.006	0.939	<0.001
Error (stimulus)	7.024	52	0.135			
Trial	9.188	1	9.188	20.726	<0.001	0.285
Trial × Group	0.089	1	0.089	0.201	0.656	0.004
Error (trial)	23.052	52	0.443			
Context × Trial	0.069	1	0.069	0.534	0.468	0.010
Context × Trial × Group	0.051	1	0.051	0.394	0.533	0.008
Error (Context × Trial)	6.691	52	0.129			
Between-subjects effects						
Group	0.082	1.00	0.082	0.435	0.513	0.008
Error	23.370	52	0.449			

Note. Results form 2 × 2 × 2 analyses of variance[1] of skin conductance responses to context transitons with Stimulus (CS+, CS) and Trial (end of extinction, start of reinstatement) as within-subject variables and Group (novel exploration, control) as between-subjects variable. SCRs are root transformed and mean range corrected.
